# Toxicological Assessment of 17β-Estradiol and 17α-Ethinylestradiol After Adsorption in a Biomass Filter Associated with the Nanomaterial δ-FeOOH

**DOI:** 10.3390/ijerph23050677

**Published:** 2026-05-20

**Authors:** Fernanda Junger Schaper, Isadora Amaral Ramos, Sthefany Burmann Soares, Alice Camilo Duarte, Edipaula Barbosa Franco, Camila de Sousa Queiroz Almeida, Cleide Aparecida Bomfeti, Jairo Lisboa Rodrigues, Márcia Cristina da Silva Faria

**Affiliations:** Instituto de Ciência, Engenharia e Tecnologia (ICET), Universidade Federal dos Vales do Jequitinhonha e Mucuri (UFVJM), Teófilo Otoni 39803-371, MG, Brazil; fernanda.schaper@ufvjm.edu.br (F.J.S.); isadora.amaral@ufvjm.edu.br (I.A.R.); sthefany.burmann@ufvjm.edu.br (S.B.S.); alice.duarte@ufvjm.edu.br (A.C.D.); edipaula.franco@ufvjm.edu.br (E.B.F.); camila.queiroz@ufvjm.edu.br (C.d.S.Q.A.); cleide.bomfeti@ufvjm.edu.br (C.A.B.); jairo.rodrigues@ufvjm.edu.br (J.L.R.)

**Keywords:** 17β-estradiol, 17α-ethinylestradiol, biomass, nanomaterial δ-FeOOH, adsorption, toxicity bioassays

## Abstract

**Highlights:**

**Public Health Relevance—How does this work relate to a public health issue?**

- Millions of women worldwide use these hormones as contraceptive methods and for hormone therapy, which highlights the continuous and abundant introduction of these compounds into the environment, both through their excretion by the human body and through the direct disposal of unused or expired medications.

**Public health significance—Why is this work of significance to public health?**

- Several studies demonstrate that these hormones exhibit variable removal efficiencies in water and wastewater treatment plants; however, even at any measurable concentration, they may still be sufficient to cause numerous adverse effects on aquatic organisms, such as changes in fertilization rates, behavioral modifications, histopathological alterations, immunosuppression, development of female sexual characteristics in males, and inhibition of sexual organ development. In humans, the earlier onset of puberty and the decline in semen quality over the last century have also emerged as central topics in this discussion, suggesting a possible relationship with exposure to these hormones.

**Public health implications—What are the key implications or messages for practitioners, policy makers and/or researchers in public health?**

- There is an increasing need for the development of technologies capable of efficiently removing these hormones from aquatic matrices while maintaining low operational costs, so that they can be considered feasible and applicable for use in water and wastewater treatment plants.

**Abstract:**

Emerging contaminants pose significant risks to ecosystems yet are not routinely included in standard monitoring or regulatory frameworks. Among these substances, endocrine disruptors such as β-estradiol and 17α-ethinylestradiol threaten both human and environmental health by interfering with metabolism, reproduction, and development across multiple species. These hormones are continuously released into the environment through excretion and improper disposal, and conventional water treatment processes are largely ineffective at removing them. As a result, they can accumulate in aquatic organisms and enter the human food chain. Recent studies have demonstrated that banana peel, *Pleurotus ostreatus* biomasses, and the nanomaterial δ-FeOOH are efficient, low-cost materials for the removal of toxic metals, suggesting their potential applicability for eliminating estrogenic compounds. Therefore, this study aimed to evaluate the removal of β-estradiol and 17α-ethinylestradiol using filters composed of banana peel and *P. ostreatus* biomass combined with δ-FeOOH. Hormone removal efficiency was assessed by LC-MS, and toxicity reduction was evaluated through bioassays. The results showed up to 100% removal of hormone concentrations and a significant decrease in sample toxicity, indicating that this filtration system represents a safe and effective alternative for removing organic contaminants from water.

## 1. Introduction

In recent years, emerging contaminants (EC) have gained increasing attention worldwide due to their detection in soil, water, and particulate matter that, despite not being included in routine monitoring programs or having specific legislation regulating their presence posing a risk to ecosystems, are not included in monitoring programs and do not have specific legislation for their control [1]. These contaminants can be of both anthropogenic origin (found in domestic, industrial, hospital effluents and those from agriculture and livestock) and of natural origin (found in different organisms). Although often present at low concentrations, their chemical stability and tendency to bioaccumulate pose significant ecological risks [2].

Among the ECs that present the most potential risk to human health and the environment are pesticides, insecticides, personal hygiene products, medicines and steroid hormones. Steroid hormones, both natural and synthetic, stand out and are notable endocrine disruptors or disruptors (ED) that interfere with the endocrine system, altering the synthesis and metabolism of natural hormones or modifying the levels of hormone receptors. These disruptions affect critical biological functions, including reproduction, embryonic development, growth and metabolism [3].

Globally, an estimated 50 to 60 million women take oral contraceptives as their primary form of contraception, with most of them being a combination of estrogens and progestins. In addition, they are also used in menopausal hormone therapy and for ovarian development [4,5].

The natural estrogen β-estradiol (E2) produced by all mammalian females has a daily estimated excretion of 2.3 to 259 micrograms/woman, entering aquatic systems primarily via domestic sewage [6]. Agricultural effluents also contribute to environmental E2 loads, which have increased despite the hormone’s relatively rapid degradation [6].

In contrast, the synthetic estrogen hormone 17α-ethinylestradiol (EE2), widely used in oral contraceptives, drugs and animal feed additives, exhibits greater persistence, higher bioaccumulation potential, and stronger estrogenic effects.

EE2 is considered the main compound responsible for endocrine disruption in aquatic organisms, such as changes in fish and other organisms that live in the water [7,8]. The extensive use of these hormones ensures their continuous and abundant release into the environment through excretion and improper disposal of unused or expired medications [9].

Despite the numerous damages they cause to ecosystems, conventional methods of water and sewage treatment plants are unable to efficiently remove these hormones, with varying levels of removal, which leads to a continuous and cumulative intake, both by aquatic organisms and by humans [10]. For instance, a study in southern Sweden reported approximately 47% removal of estradiol by WWTPs [11], while research in Ulsan, South Korea, revealed seasonal variation in pharmaceutical removal efficiency, depending on treatment processes [12].

Even at trace concentrations, these hormones can induce adverse effects in aquatic organisms, such as changes in fertilization rates, behavioral changes, development of female sexual characteristics in males, and inhibition of the development of sexual organs [13,14]. Although there are few studies in Brazil on the occurrence of estrogens in aquatic matrices, available data indicate substantially higher concentrations compared to international reports, reaching up to 5000 ng L^−1^ in raw sewage and 4000 ng L^−1^ in surface waters [15].

Several countries have implemented regulations to limit these hormones in water bodies [7]. For example, the Australian Guidelines for Water Recycling, created by two Australian councils, recommends maximum drinking water values of 175 ng/L for E2 and 1.5 ng/L for EE2 [16]. The European Community suggests even lower limits for surface waters, 0.4 ng/L for E2 and 0.035 ng/L for EE2 [17]. The Convention for the Protection of the Marine Environment of the North-East Atlantic (OSPAR) has also included these two hormones in the list of substances of concern due to possible toxicity, persistence, and bioaccumulation, posing a risk to the reproduction of fish and other aquatic animals [18].

Advanced treatment methods such as biological degradation and chemical oxidation have been explored for hormone removal [19,20], yet these approaches often require high energy inputs, costly equipment, and may generate toxic byproducts, limiting their sustainability [21].

According to Prokić et al. [22], adsorption is considered one of the most efficient and cost-effective processes for the removal of environmental contaminants, and the choice of the appropriate adsorbent is the key element in the application of this method. Different materials have already been used as adsorbents for the removal of steroid estrogens from water, such as: chitin, chitosan, activated carbon, clayey hybrid materials, graphene magnetic oxides, cyclodextrin polymers and iron nanoparticles.

Specifically, δ-FeOOH nanoparticles have demonstrated chemical stability, ferromagnetic properties, ease of dispersion in water, and simplicity and low cost of synthesis, making them attractive adsorbents, especially for toxic metals like arsenic [23]. In addition, the synthesis of this nanoparticulate material is simple and low-cost, as are natural or biological adsorbents from banana peel biomass and the *Pleurotus* fungus, which have a great potential for removing organic compounds due to the large surface area and interaction with functional groups [24].

Recent studies have demonstrated that banana peel biomass associated with the nanomaterial δ-FeOOH is a low-cost and efficient method for removing toxic metals from water, being a promising alternative for the removal of estrogen hormones in water and sewage treatment plants [25].

However, due to the increased use of nanomaterials in various studies and their high capacity to disperse in the environment, there is a need to evaluate their potential risks through bioassays, establishing the toxicological profile of these materials for a better understanding of their interactions with living organisms, in addition to verifying their efficiency in removing hormones [26]. Despite the growing concern over the presence of steroid hormones in aquatic environments and the documented inefficiency of conventional wastewater treatments [11,12], there remains a significant gap in the development of low-cost, sustainable, and ecologically safe alternatives for their removal—especially in tropical and developing regions such as Brazil.

Therefore, this study aims to evaluate the toxicity of water samples contaminated with β-estradiol and 17α-ethinylestradiol after treatment by adsorption using banana peel biomass filter and *Pleurotus* biomass associated with the nanomaterial δ-FeOOH. This work intends to verify the effectiveness and environmental safety of this combined adsorption approach as a viable alternative for removing steroid hormones from aquatic environments.

## 2. Materials and Methods

### 2.1. Place of Study

The synthesis of the nanomaterial δ-FeOOH, preparation of banana peel and *Pleurotus ostreatus* biomass, construction of the filters, preparation of sample groups, and in vitro bioassays were all conducted at the Laboratories of Contaminant Analysis and the Multi-User Laboratory of ICET-UFVJM, Campus do Mucuri, Teófilo Otoni-MG.

### 2.2. Group of Samples

The samples were prepared using ultrapure Milli-Q water (resistivity 18.2 MΩ cm) obtained using the Direct-Q^®^ 3 UV Remote Water Purification Systems. The 17β-estradiol (Sigma-Aldrich, St. Louis, MO, USA, purity ≥ 97%) and 17α-ethinylestradiol (Sigma-Aldrich, purity ≥ 98%) standards were diluted to 200 μg/L in 10% dimethyl sulfoxide (DMSO, Exodus Científica, Sumaré, SP, Brazil). The concentration values of the hormones were obtained based on the linear ranges used in other studies [27].

Milli-Q water was used as a negative control (C−) and the 4 × 10^−4^ M methyl methanesulfonate (MMS) was used as a positive control (C+), obtained from Sigma-Aldrich (St. Louis, MO, USA), CAS No 66-27-3, a monofunctional alkylating agent, widely used as an inducer of deoxyribonucleic acid (DNA) damage.

The E2 and EE2 samples were submitted to the developed filters, allowing a comparison of the adsorption and removal efficiency of the compounds with the NC and with the initial dosages of the hormones, as described in Table 1.

### 2.3. Synthesis of δ-FeOOH

The synthesis of δ-FeOOH was performed as described by Faria et al. [23], by precipitation of Fe^2+^ (ferrous ion) by adding 400 mL of aqueous solution of NaOH at 2 mol·L^−1^ (equivalent to 32 g NaOH) in a 400 mL solution containing 44,483 g Fe(NH_4_)_2_(SO_4_)2·6H_2_O, supplied by Sigma Aldrich (Darmstadt, Germany), already heated to 60 °C. Following the formation of a green precipitate, 20 mL of 30% (*v*/*v*) hydrogen peroxide (H_2_O_2_) was immediately added under constant agitation for 30 min, resulting in the formation of reddish-brown δ-FeOOH. The solution was allowed to settle for 48 h, after which the supernatant was removed and the pH was checked. The precipitate was washed in ultrapure water and allowed to settle until the pH reached neutrality. Subsequently, the precipitate was transferred to Falcon tubes and centrifuged at 6000 rpm for 10 min, and the supernatant was removed. The tubes were covered with Parafilm, some holes were drilled and stored at −70 °C before going to the freeze-drying for 24 h to remove all moisture. Finally, the dried precipitate was crushed and macerated in a mortar before being stored in Falcon tubes.

### 2.4. Bio-Nano-Technological Filter Assembly

**Figure 1:**
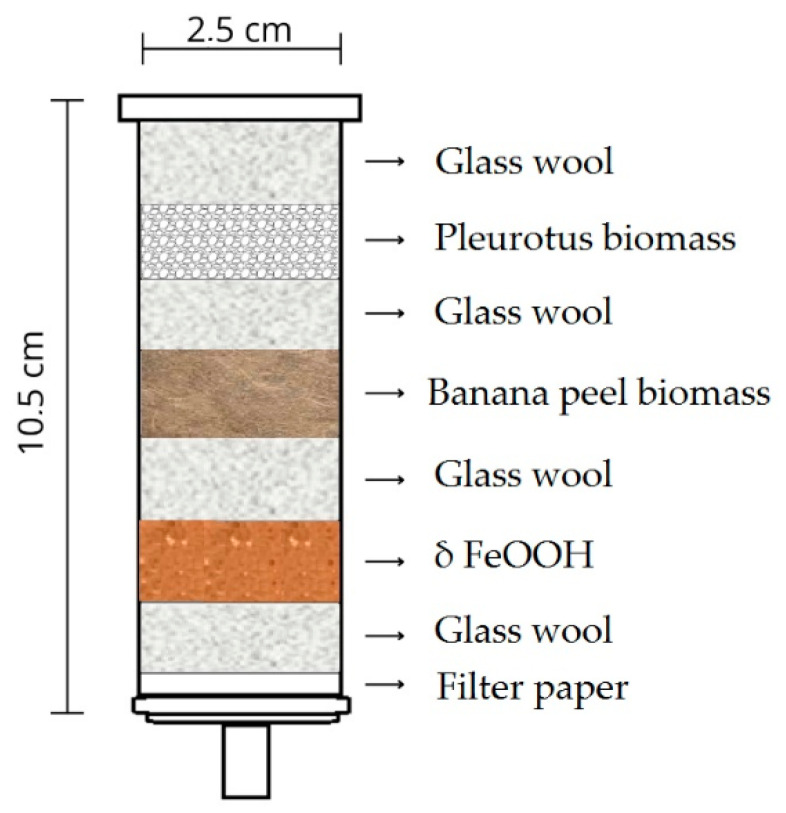
Representation of the assembly of nanobiotechnological filters.

The filters were developed using 20 mL syringes to facilitate the flow of contaminated samples. They were assembled in the following configuration: a first layer of filter paper; a second layer of glass wool to prevent the components from passing through the syringe orifice; and a third layer consisting of either the nanomaterial δ-FeOOH, banana peel biomass, or *Pleurotus* biomass. Additionally, combined filters were prepared to evaluate the synergy between the materials. These contained the nanomaterial δ-FeOOH, banana biomass, and *Pleurotus* biomass in the same quantities used in the individual filters. All components were interspersed with glass wool and filter paper near the orifice, as illustrated in Figure 1.

The syringes used in the filter assembly were washed in 10% Extran solution, rinsed three times in distilled water, and dried in an oven at 50 °C for 24 h.

Beakers, watch glasses, spatulas, tweezers, and other utensils were sterilized in an autoclave following standard procedures.

The banana peel biomass was obtained using the methodology of Santos et al. [28], where the peel was washed with ultrapure water, placed in an oven for drying at 70 °C, and ground in an industrial blender SHOP6301. After grinding, the obtained product was sieved for Bertel particle size analysis using a 40 mesh. These biomasses were then lyophilized and subsequently stored under appropriate conditions to preserve their properties.

The methodology for preparing *Pleurotus* biomass was adapted from Babá et al. [29] and Buratto et al. [30]. Cultivation was performed in 500 mL Erlenmeyer flasks containing 200 mL of Potato Dextrose (PD) medium, sterilized by autoclaving at 121 °C for 15 min. Under aseptic conditions in a laminar flow hood, twenty 1 cm diameter disks of *Pleurotus* sp. Mycelium, previously cultured on Potato Dextrose Agar (PDA), was inoculated into the liquid medium. The flasks underwent static incubation for 15 days. Following this period, the mycelium was separated from the medium, rinsed with distilled water, and dehydrated at 70 °C until a constant weight was achieved. Finally, the dry biomass was pulverized in a blender and stored in sealed flasks at 70 °C.

For the preparation of the filters, a standardized mass of 0.88 g was used for both δ-FeOOH and the biomass materials. This value was adopted based on the findings of Gonçalves [31], which demonstrated superior adsorption performance at this mass.

For the filtration process, a 30 mL volume was used for each sample. The samples were allowed to drain by gravity through the filters, which were secured to a universal support. The filtered samples were then stored in amber glass at 4 °C until use.

### 2.5. Identification and Quantification of Estrogens

The detection of E2 and EE2 hormones was performed by liquid chromatography associated with Shimadzu model 8050 (Kyoto, Japan) triple-quadrupole mass spectrometry associated with Liquid Chromatography—Mass Spectrometry (LC-MS). This method is advantageous for analyzing such compounds, as it eliminates the extensive sample preparation required by other techniques while offering very high sensitivity, enabling the identification of hormones at concentrations below parts per billion.

Additionally, the LCMS-8050 can acquire both qualitative and quantitative information in a single analysis [32]. The analytical process was carried out in partnership with Shimadzu.

Sample preparation involved filtering the samples through a 0.45 μm Polyvinylidene Fluoride (PVDF) filter prior to injection, after which the filtered volume was transferred to a capped vial.

For the analysis of the intended hormones, the parameters are presented in Table 2 for Chromatographic conditions and Mass spectrometer conditions.

**Figure 2:**
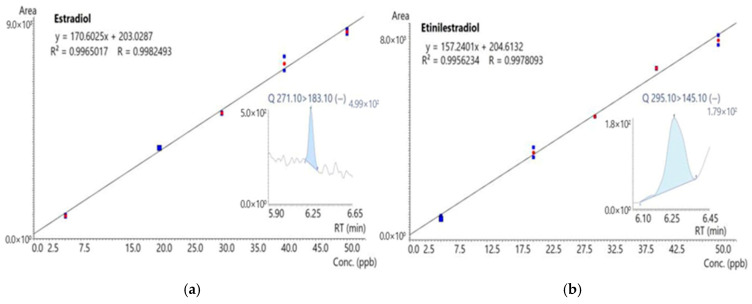
Calibration curves performed with hormones E2 (**a**) and EE2 (**b**).

The calibration curves were prepared and analyzed in duplicate over a concentration range of 5 to 50 μg/L. Figure 2 presents the curves for 17β-estradiol and 17α-ethinylestradiol, both of which showed excellent linearity (R^2^ > 0.99 and R > 0.99).

### 2.6. Bioassays

#### 2.6.1. *Allium cepa* Test

To evaluate toxicity, the *Allium cepa* (onion) test was performed on samples contaminated by hormones before and after filtration, as described and adapted from previous studies [33]. In short, the bioassays were performed in triplicate. In each replicate, 100 *Allium cepa* seeds (Baia Periforme variety, Isla^®^, Porto Alegre, RS, Brazil) were cultivated in Petri dishes lined with filter paper moistened with ultrapure water.

The seeds were incubated for 5 days at 25 °C and until the roots reached approximately 2 cm in length. Roots were then excised and fixed in Carnoy’s solution (3 ethanol:1 acetic acid) in 1.5 mL microtubes for 6 h. Carnoy’s solution was replaced by 1 mL of 70% ethanol for another 6 h and stored until further analysis.

For microscope slide preparation, the roots were washed three times with ultrapure water and subjected to acid hydrolysis with 1 M HCl solution at 60 °C for 11 min. After acid hydrolysis, the roots were transferred to 1.5 mL microtubes containing Schiff’s Reagent (Merck, Rahway, NJ, USA) and kept in dark for 2 h. Next, individual roots were sectioned, placed on glass slides, and stained with 2% acetic carmine. After 8 min, a coverslip was applied, and the preparations were observed under a light microscope to detect chromosomal alterations indicative of genotoxicity. The microscopic parameters are performed from the meristematic cells of the onion root and focus on three types of analyses: the presence of micronuclei (MN), indicating mutagenicity [34]; Mitotic Index (MI) calculated as the ratio between the number of cells in mitotic division and the total number of cells observed, determining cytotoxicity; and chromosomal aberrations (CA).

#### 2.6.2. Comet Assay

The comet assay was performed following the protocol of Singh et al. [35], with adaptations from Yildiz et al. [36] and Oliveira et al. [37].

To perform the Comet assay, *Allium cepa* seeds were first germinated in ultrapure water, following the methodology previously described for the *Allium cepa* Test. After five days of incubation at 25 °C, 1 mL of each treatment was added to the Petri dishes containing the germinated seeds: positive control (methyl methanesulfonate, MMS 4 × 10^−4^ mol/L), negative control (ultrapure water), and filtered samples. The seeds remained under these conditions for an additional 24 h at 25 °C.

Microscope slides were washed with 10% Extran for 60 min, rinsed with distilled water, and dried at 50 °C. After drying, the slides were stored in 70% ethanol in the refrigerator. All subsequent slide preparation was performed under no-light conditions. Each slide was covered with a thin layer of 1.5% normal melting point agarose and dried at room temperature overnight. For each sample/slide, the apical 1 cm tip of 20 roots was excised on a Petri dish above permanent ice. Next, 1 mL of 85% lactic acid was added for 1 min. Then, the roots were washed twice with 1 mL of the 1X PBS solution. The roots were dried on filter paper and transferred to a 2.0 mL microtube. In each microtube, 200 μL of 1X PBS was added, and the roots were chopped with a scalpel for two minutes. The solution formed with the meristematic cells was then transferred to a new 2.0 mL microtube without taking the carcasses, keeping them in the refrigerator until all samples were finalized.

For embedding, we added 200 μL of 1% low melting point agarose to each cell suspension, homogenized gently, and pipetted it onto the pre-coated slides. Shortly after this step, we covered it with a large coverslip. For each treatment, we analyzed three slides of 20 roots.

Once the slides were completely dry, the coverslips were carefully removed, and the slides were placed in the electrophoresis tank filled with alkaline buffer (1 mM Na_2_EDTA, 300 mM NaOH, pH > 13) for an initial incubation period of 20 min. Subsequently, electrophoresis was performed at 25 V for 10 min. Following the electrophoresis, the slides were washed in 0.4 M Tris-HCl (pH 7.5) three times for 5 min, fixed in cold methanol for another 5 min, and dried at room temperature overnight. The dried slides were stored in the refrigerator until staining.

Each slide was stained with 100 μL of freshly prepared 20 μg/mL ethidium bromide for 5 min. Slides were then covered with coverslips and examined using an epifluorescence microscope (Olympus BX60, Essex, UK) with a UV filter. The images were then captured and saved to proceed with the analyses in the CASP domain software (IS Capture) (version 5.2). The Image J 1.52t software was used and the photographic protocol used consisted of a scale of 6.29 pixels per micrometer, according to studies by Brianezi et al. [38]. For each treatment, 100 randomly selected nucleoids were visually classified based on tail fluorescence intensity and scored from 0 to 4, resulting in a total slide score range of 0–400 arbitrary units (AU), according to Yildiz et al. [36].

The comet tails were assessed using Damage Index (DI) and Damage Frequency (DF%). The DI was calculated as the sum of the products of the multiplication between the number of comets of each class by the denominator digit of that class (0, 1, 2, 3, 4). In this way, the DI of each group could vary between 0 (no damage) and 400 (maximum damage). The DF% was calculated as the percentage of all damaged comets (class 1 to class 4) in relation to the total number of comets counted, ranging from class 0 to class 4 (total number).

### 2.7. Statistical Analyses

Statistical analyses were conducted using the GraphPad Prism 10.4.1 software. Results are presented as Mean ± Standard Error of the Mean (SEM) and are representative of at least three independent experiments. Comparisons between groups were performed using the Student’s *t*-test, the non-parametric Mann–Whitney test for both the *Allium cepa* Bioassay and the Comet Assay. Differences were considered significant when *p* < 0.05.

## 3. Results

### 3.1. LC-MS Analysis

The concentration results for the analytes of interest are presented in Table 3. It is noteworthy that there was no detection of the carry over effect (memory effect) in any of the injections performed.

### 3.2. Toxicological Analysis

#### 3.2.1. *Allium cepa* Test

The macroscopic analysis was conducted descriptively, evaluating both the germination percentage and the average root length to assess the toxicity of the hormone-contaminated samples before and after filtration (see Table 4). Notably, the unfiltered E2 and EE2 solutions at a concentration of 200 μg·L^−1^ exhibited reduced germination rates (51%) and markedly lower average root lengths of 0.96 ± 0.35 cm and 1.17 ± 0.42 cm, respectively.

For E2, the filter with all the adsorbent materials in the study demonstrated greater efficiency in relation to germination taxa, with 83% of the seeds germinated in sample A8. While for EE2, the samples that had the highest germination rates were through the filter composed only of δFeOOH (A11) and the filter composed of *Pleurotus* biomass (A10), with 79% of germinated seeds for both.

The results of the Mitotic Index (MI) are presented in Table 4 for E2 and EE2. Accordingly, the Mann–Whitney test, we observed statistically significant differences in the water samples after going through the filtration process when compared to the negative control (H_2_O).

**Figure 3:**
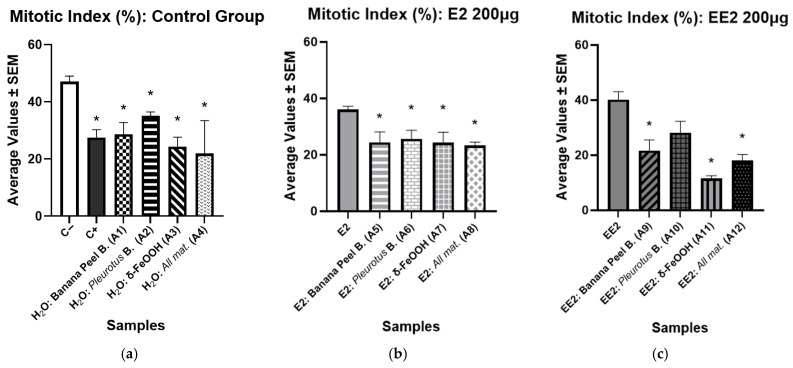
Results of the mean values ± SEM obtained in the study of the mitotic index (MI) of the samples after the filtration process, with a significant difference considering *p* < 0.05 (*): (**a**) Negative Control; (**b**) E2 200 μg; (**c**) EE2 200 μg.

The results of the evaluation of the presence of cytotoxicity are shown in Figure 3. The Mann–Whitney test revealed significant differences at *p* < 0.05. The positive control (MMS) and the water samples after filtration were compared to the negative control (H_2_O). The results showed a decrease in the mitotic index in the meristematic cells of onions subjected to the filtration process.

In Figure 3b, which presents the results of the samples containing E2 after filtration, a significant reduction is observed, when *p* < 0.05, in the Mitotic Index in all filters, when compared to E2 at the initial concentration of 200 μg (36.08 ± 1.16). The lowest value recorded was 23.26 ± 1.25, observed in the filter that used all combined materials (A8). The same occurred for the EE2 samples after filtration (Figure 3c), in which the filters used showed a significant reduction in the Mitotic Index in relation to the initial concentration of the hormone, which presented a MI of 40.22 ± 2.86.

**Figure 4:**
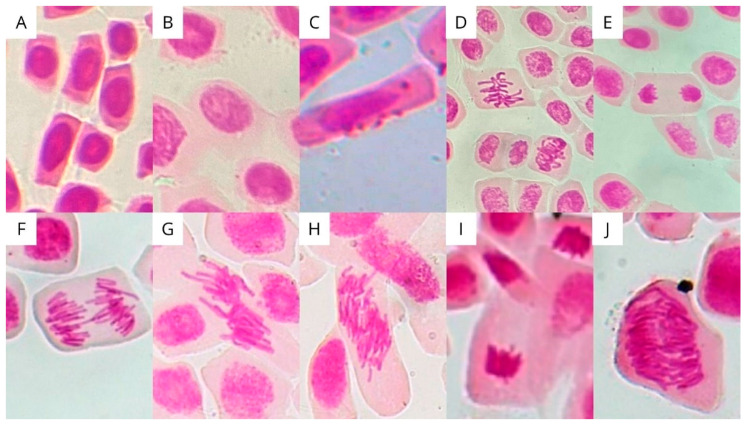
Photomicrograph of cell divisions, chromosomal alterations and micronuclei observed in *Allium cepa* cells. Cells in (**A**) interphase; (**B**) prophase; (**C**) micronuclei; (**D**) metaphase; (**E**) anaphase; (**F**) Anaphase with chromosomal bridge; (**G**) Metaphase with adhesion; (**H**) Polyploid metaphase; (**I**) Telophase; (**J**) Polyploid anaphase.

**Figure 5:**
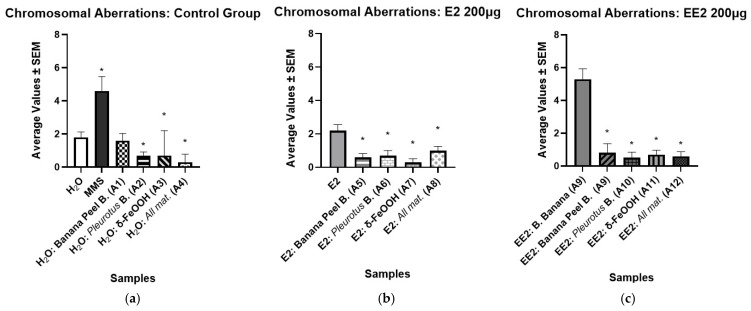
Results of the mean values ± SEM obtained in the study of chromosomal aberrations (CA) of the samples after the filtration process, with a significant difference considering *p* < 0.05 (*): (**a**) Negative Control; (**b**) E2 200 μg; (**c**) EE2 200 μg.

Table 4 summarizes the results of the chromosomal aberration analyses for the filtered samples of E2 and EE2, respectively. The anomalies identified include: loss, breakage, adhesion, bridging, binucleate cells, c-metaphase, and polyploid (see Figure 4). The most frequent anomalies in both E2 and EE2 samples were bridges and binucleates. Figure 5 shows the arithmetic means and the standard error of the mean chromosomal alterations for each of the samples.

A significant reduction in chromosomal aberrations (CAs) was observed in the water samples filtered through the A4 filter, which was composed of all the tested materials (see Figure 5a). A similar decrease in CA frequency was also noted in the filter composed only of δ-FeOOH nanomaterial and Pleurotus biomass, indicating reduced genotoxicity compared to unfiltered water samples. We found a significant decrease in CA after the E2 filtration in A5, A6, A7 and A8, suggesting an effective reduction in genotoxic potential relative to the unfiltered E2 solution (see Figure 5b). Similarly, EE2 hormone also showed a significant CA decrease in all filters used (see Figure 5c).

**Figure 6:**
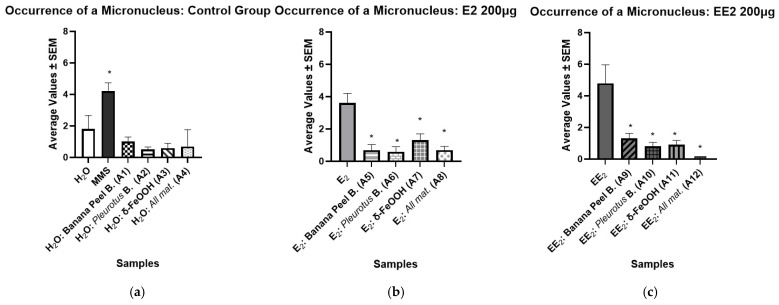
Results of the mean values ± SEM obtained in the study of the presence of micronuclei (NM) in the samples after the filtration process, with a significant difference considering *p* < 0.05 (*): (**a**) Negative Control; (**b**) E2 200 μg; (**c**) EE2 200 μg.

The presence of micronuclei (MN), cytoplasmic chromatin bodies resulting from acentric chromosomal fragments that fail to integrate into the daughter nuclei during mitosis, was used as an indicator of mutagenic potential [39]. Table 4 and Figure 6 show significantly low mean values of micronuclei in the water samples after being filtered, suggesting that the tested filters did not induce mutations in the water samples (see Figure 6a). In contrast, the positive control (MMS) exhibited a markedly higher MN frequency, consistent with its known DNA-damaging capacity (see Figure 6a). Regarding the samples containing the hormone E2 (see Figure 6b), the Mann–Whitney test revealed a statistically significant reduction in the frequency of micronuclei (*p* < 0.05) after filtration, indicating a decrease in mutagenic activity. Similarly, the samples containing the hormone EE2 also showed a significant reduction in micronuclei formation following the filtration process (see Figure 6c).

#### 3.2.2. Comet Assay (Results)

Genotoxicity was also evaluated using the comet assay for controls and samples containing E2 and EE2 hormones before and after filtration (Table 5).

**Figure 7:**
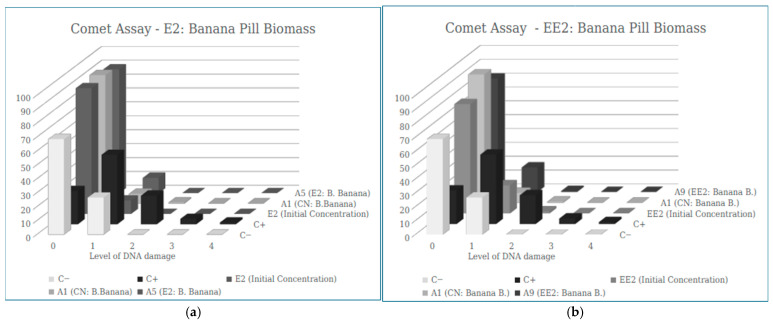
Results of the mean values ± SEM of the Damage Index (DI) obtained in the Comet assay in the sample of E2 (**a**) and EE2 (**b**) after the filtration process by the banana peel biomass.

Figure 7 presents the distribution of the different classes of DNA damage identified in the Comet assay, comparing the controls, unfiltered hormone solutions (E2 and EE2), and the same hormones after filtration through the various filter types tested. It is noted that there is a lower number of cells for damage class 1, 2, 3 and 4 in all filtered samples when compared to the positive control, while an increase in the number of cells in score 0 is observed, indicating that the adsorbent materials used did not increase the genetic damage in the cells after filtration, for both E2 and EE2.

**Figure 8:**
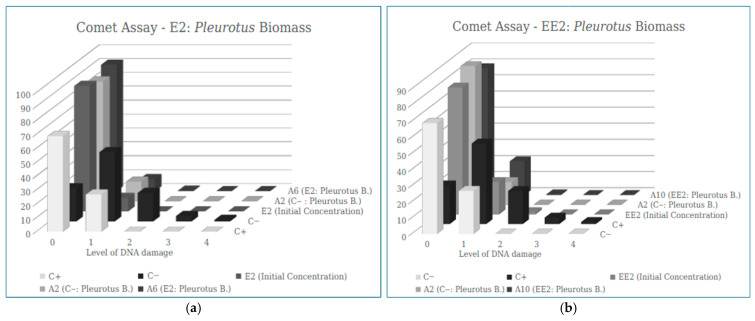
Results of the mean values ± SEM of the Damage Index (DI) obtained in the Comet Assay in the (**a**) sample of E2and (**b**) sample of EE2 after the filtration process by Pleurotus Biomass.

In the filter developed from *Pleurotus* biomass, the results obtained show that the samples containing E2 (see Figure 8a) and EE2 (see Figure 8b), after passing through the filter, did not have an increased number of cells with damage, as occurred in the positive control, indicating that there is no genotoxic potential in this adsorbent material.

**Figure 9:**
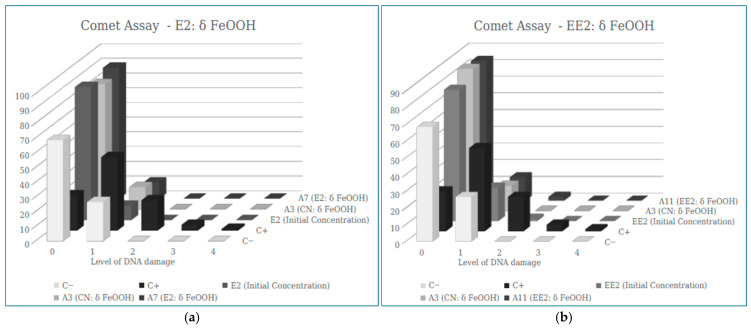
Results of the mean values ± SEM of the Damage Index (DI) obtained in the Comet Assay in the (**a**) sample of E2 and (**b**) sample of EE2 after the filtration process by the nanomaterial δ-FeOOH.

Regarding the filter developed from δ-FeOOH for E2 removal, the absence of damage of greater intensity (damage 2, 3 and 4) is notable in sample A7, with results of the average ID values (9.3 ± 4.33) much lower than the positive control (108.67 ± 18.70), showing that the nanomaterial is safe in relation to genotoxic potential (see Figure 9a).

For the removal of EE2, sample A11 also did not show genotoxic potential, with an ID value of 19.00 ± 3.61 (see Figure 9b).

**Figure 10:**
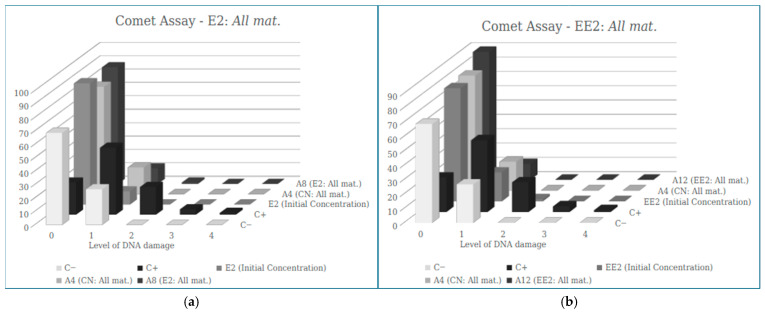
Results of the mean values ± SEM of the Damage Index (DI) obtained in the Comet assay in the (**a**) sample of E2 and (**b**) sample of EE2 after the filtration process by all adsorbent materials of this study (All mat.).

In Figure 10, the results obtained show that the samples containing E2 and EE2 maintained a low DI even after passing through the filter composed of all adsorbent materials, indicating that there was no presence of genotoxicity.

The filter developed for E2 removal with all the adsorbent materials in the study (A8) showed a significantly lower ID than that found in the Positive Control. This corroborates the damage analysis, which shows high values of zero damage (without any DNA alteration) in the sample, unlike the positive control, which reveals lower values of zero damage (see Figure 10a).

For EE2 removal, the cell ID values found in sample A12 (11.00 ± 5.57) were significantly lower than the ID of the positive control (108.67 ± 18.70), with a higher number of cells scoring 0 than in the positive control (see Figure 10b).

It can also be observed that no significant difference was observed in the damage scores between the water control groups and the samples after filtration, suggesting that the adsorbent materials used in this study are not determining factors in the extent of DNA damage.

## 4. Discussion

In general, the results regarding the adsorption capacity of the filters used in this study for the removal of hormones E2 and EE2 showed concentration values below the detection limit of the equipment, strongly indicating the efficiency of the adsorbents used.

Table 6 presents a comparison of the results found in this study for the removal of E2 and EE2 with other adsorption studies reported in the literature for the removal of these hormones.

It is evident that the maximum removal values achieved in this study (100% for E2 and EE2) were higher than the values previously reported. Similarly, Alhares et al. [40] reported 100% adsorption efficiency using rice husk biomass coated with copper oxide nanoparticles, highlighting a cost-effective and environmentally friendly method to remove estrogens from aqueous media. That study also emphasizes that adsorption parameters significantly influence the removal process, underscoring the importance of investigating aspects related to equilibrium isotherm and absorption kinetics.

According to Aquino et al. [48], endocrine disruptors with log Kow < 2.5 are highly hydrophilic and show low affinity for biomass. For those with log Kow between 2.5 and 4.0, a moderate uptake trend is expected in these matrices, such as EE2. EDs with log Kow > 4.0 are highly hydrophobic and have a great potential to be adsorbed. These characteristics help explain the high rate of hormone removal, since the log Kow of E2 and EE2 are 4.01 and 3.67, respectively.

**Figure 11:**
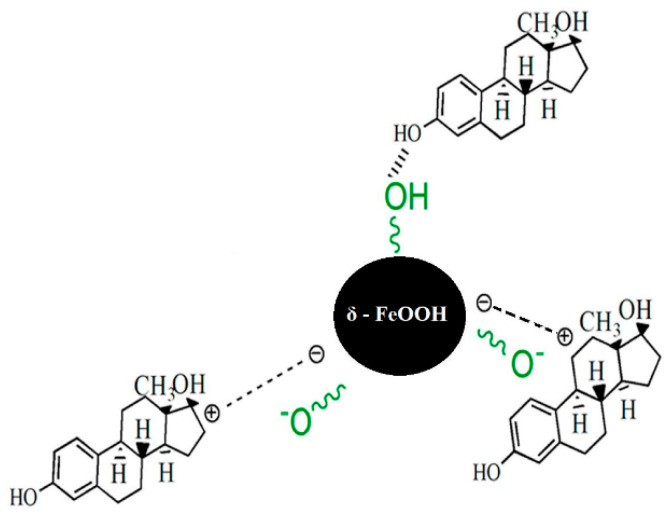
Representation of the chemisorption mechanism involving the nanomaterial and the hormones E2 and EE2.

Mpupa et al. [49] reported that the adsorption process between iron oxyhydroxide nanorods (akaganeite, β-FeOOH) and the hormone β-estradiol occurs homogeneously at the surface sites of the β-FeOOH nanomaterial and that β-estradiol adsorption can be assumed to occur in a monolayer fashion. Furthermore, the authors suggest that the adsorption process was driven by chemisorption involving the electrostatic interaction between the positively charged adsorbent and lone pairs of β-estradiol electrons (see Figure 11).

Georgin et al. [50] highlighted that materials such as graphene oxides, nanocomposites, and carbonaceous materials are frequently used in adsorption processes. Medium pH, particularly under acidic to neutral conditions, affects efficiency, and the ambient temperature favors the process. Investigating these parameters is essential to optimizing adsorption performance.

The same author confirmed the exothermic nature of most adsorption processes via thermodynamic analysis, indicating that physical interactions predominate. This supports the feasibility of reusing materials, potentially lowering operational costs.

The results reveal decreased mitotic index (MI) values, which may be attributed to the cytotoxic effects caused by the contact of water with certain bioactive compounds present in the adsorbents. Some of these compounds may be leached into the water during filtration, impairing plant cell division [51,52]. Additionally, fungi of the genus *Pleurotus* can produce several secondary metabolites, which, when interacting with dividing cells, inhibit or delay mitosis [53]. Regarding δ FeOOH, the decrease in the mitotic index observed in roots exposed to ultrapure water may be associated with the generation of reactive oxygen species or the presence of residual nanoparticles, according to some studies that investigated the toxic effects of metallic nanomaterials [54,55].

According to Merki-Feld, Seeger and Mueck [56], both E2 and EE2 cause a significant increase in the rate of cell proliferation, which may explain the higher MI values for the hormones at their initial concentration when compared to filtered samples.

Similarly, Yager [57] states that E2 increases cell proliferation through estrogen receptor-mediated signal transduction, which corroborates the higher MI of the hormone before passing through the filter.

Several studies suggest that the hormones E2 and EE2 are genotoxic, which is justified due to the mechanism of action of these substances that occurs through the interaction with estrogen receptors located in the cytoplasm and cell nucleus, altering the conformation of these receptors and consequently interacting in the synthesis of mRNA, resulting in mutagenic effects [6].

According to the results obtained in the study by Rodrigues et al. [58], genotoxicity can be caused indirectly by EE2-induced oxidative stress. The inefficiency of antioxidant defense pathways can result in oxidative stress, which culminates in molecular damage to cellular macromolecules of great importance, such as DNA, proteins, and cellular lipids.

Our findings are consistent with Micael et al. [59], who demonstrated that EE2 induces genotoxic effects in zebrafish, after exposure to 3.5 ng/L for 4 months. Similarly, Rodrigues et al. [58] showed genotoxic disturbances after exposure to EE2, confirming that 17α-ethinylestradiol represents a potential chemical pollutant.

Petridis et al. [60] tested the genotoxic effects of E2 and EE2 in plant species, hypothesizing a possible link to intersex traits. Their findings partially supported this, showing genotoxicity only at concentrations above those typically found in freshwater environments.

Despite the numerous advances that nanomaterials have achieved, some of them can have toxic effects on aquatic organisms when in high concentrations, and can even cause oxidative stress, membrane destruction and cell death due to the loss of essential vital functions. In addition, nanomaterials can reduce the growth and reproduction rate of plants, as well as reduce the photosynthetic efficiency of plant cells and alter the content of plant pigments [61]. Therefore, toxicological assessments are crucial to better understand interactions between nanomaterials and aquatic environments, ensuring their safe use.

In comparing the two genotoxicity assessment techniques used, *Allium cepa* and the Comet assay, it becomes clear that they are complementary. While the Comet Test detects DNA strand breaks linked to apoptosis, the *Allium cepa* Test identifies chromosomal aberrations in cells, offering a broader view of genotoxic mechanisms. This leads to a classification of different genotoxicity evaluation endpoints, emphasizing the importance of maintaining a combination of tests for better discernment of the mutagenicity of agents and evaluation of low doses of effects.

## 5. Conclusions

The presence of substances with estrogenic potential in the environment has been widely reported by researchers worldwide. However, information regarding the adverse effects of these substances on different organisms remains limited, particularly with respect to threshold concentrations of concern.

Given the toxicity of the hormones E2 and EE2, and the ineffectiveness of conventional wastewater treatment plants in removing these emerging contaminants, this study presents a filtration system capable of removing such substances from contaminated water samples. The system uses adsorption techniques with banana peel biomass, *Pleurotus* biomass, and the nanomaterial δ-FeOOH as adsorbent materials.

The combination of biomass with nanotechnology offers promising advances in the field of environmental remediation. A major advantage of using banana peel and *Pleurotus* biomass as adsorbents lies in their abundance and easy availability as waste materials. The use of ferroxyhyte (δ-FeOOH) nanoparticles adds further benefit due to their low cost, large surface area, and magnetic properties, which enable reuse.

The results demonstrated approximately 100% efficiency in the removal of E2 and EE2 hormones across all filters tested. Since treatment techniques often show higher efficiency at elevated contaminant concentrations, it is necessary to investigate the system’s performance at concentrations similar to those found in environmental samples (ng·L^−1^) to assess its real-world effectiveness.

Analytical methods such as liquid chromatography coupled with mass spectrometry are essential for the detection and quantification of microcontaminants in the environment. Additionally, biological assays—both in vitro and in vivo—provide valuable data on the effects of these substances on organisms, serving as an important complement to chemical analysis.

The results of the toxicological tests on *Allium cepa* presented in this study demonstrated lower values of the genotoxic and mutagenic potential of the filtered samples when compared to the initial concentration of the hormones, which demonstrates the efficiency of the adsorbent materials used. Although these compounds have the potential to cause adverse effects on aquatic biota, the concentrations employed in the toxicological bioassays were higher than those typically detected in environmental matrices. Therefore, further investigations assessing a broader range of environmentally relevant concentrations, as well as the chronic effects associated with prolonged exposure to these contaminants, are still required.

Future work should include batch adsorption experiments to obtain equilibrium and kinetic data in liquid systems. This understanding is crucial for optimizing water treatment processes and ensuring consistent process efficiency.

## Figures and Tables

**Table 1:** Groups of samples after filtration.

Filters	Sample	Sample No.
Banana peel biomass	C− (Milli-Q Water)	A1
*Pleurotus* Biomass	C− (Milli-Q Water)	A2
δ-FeOOH	C− (Milli-Q Water)	A3
All mat. ^1^	C− (Milli-Q Water)	A4
Banana peel biomass	17β-estradiol	A5
*Pleurotus* Biomass	17β-estradiol	A6
δ-FeOOH	17β-estradiol	A7
All mat. ^1^	17β-estradiol	A8
Banana peel biomass	17α-etinilestradiol	A9
*Pleurotus* Biomass	17α-etinilestradiol	A10
δ-FeOOH	17α-etinilestradiol	A11
All mat. ^1^	17α-etinilestradiol	A12

^1^ All adsorbent materials from the study.

**Table 2:** Chromatographic and mass spectrometer conditions of the LCMS 8050 for the separation of E2 and EE2 hormones.

Specifications	Aspects
ShimPach Velox, 100 mm × 3 mm × 2.7 μm (PN: 227-32010-03)	Column
A: Água + 0.15 nM de Ammonium Fluoride	Mobile Phase
B: Methanol	
Initial: 5%B → 0.5 min: 15%B → 5 min: 95%B → 7 min: 95%B → 7.1 min: 5%B → 9 min: Stop	Gradient
9 min	Total Time
0.30 mL/min	Flow
45 °C	Column Temperature
2 mm	Probe
ESI^−^	Interface
−3 kV	Interface Voltage
3 mL/min	Nebulizing Gas Flow
15 mL/min	Heating Gas Flow
350 °C	Interface Temperature
602 °C	Desolvation Temperature
250 °C	DL ^1^ Temperature
400 °C	Heat Block Temperature
3 mL/min	Drying Gas Flow

^1^ Desolvation Line.

**Table 3:** Removal of the evaluated analytes (E2 and EE2), after filtration of standard solution of 200 μg/L, in each type of filter developed, with different compositions of adsorbent materials.

Removal (%), in Standard Solution of 200 μg/L		Filter Composition (Adsorbent Material)
E2	EE2	
100%	100%	A1 (C−: Banana Peel B.)
100%	100%	A2 (C−: *Pleurotus* B.)
100%	100%	A3 (C−: δ FeOOH)
100%	100%	A4 (C−: All mat. ^1^)
99.61%	100%	A5 (E2: Banana Peel B.)
97.90%	100%	A6 (E2: *Pleurotus* B.)
100%	100%	A7 (E2: δ FeOOH)
100%	100%	A8 (E2: All mat. ^1^)
100%	100%	A9 (EE2: Banana Peel B.)
100%	100%	A10 (EE2: *Pleurotus* B.)
100%	97.97%	A11 (EE2: δ FeOOH)
100%	100%	A12 (EE2: All mat. ^1^)

^1^ All adsorbent materials from the study.

**Table 4:** Germination, Average Length, Mitotic Index, Percentage of Chromosomal Aberrations and Micronuclei Average of the control groups, E2 and EE2 samples after filtration, according to the root growth of *A. cepa* seeds.

Average MN ^4^ ± SEM ^1^	CA ^3^ % ± SEM ^1^	Mitotic Index ± SEM ^1^	Average Length (cm) ± SEM ^1^	Germination [%]	Groups
1.8 ± 0.87	1.80 ± 0.33	47.08 ± 1.97	2.47 ± 0.89	63%	Negative Control
4.2 ± 0.53	4.60 ± 0.87	27.50 ± 2.76	2.85 ± 0.87	72%	Positive Control
3.6 ± 0.60	2.20 ± 0.36	36.08 ± 1.16	0.96 ± 0.35	51%	E2 200 µg
4.8 ± 1.16	5.30 ± 0.63	40.22 ± 2.86	1.17 ± 0.42	51%	EE2 200 µg
1.0 ± 0.30	1.60 ± 0.43	28.62 ± 4.16	1.25 ± 0.78	71%	A1 (C−: Banana Peel B.)
0.5 ± 0.17	0.70 ± 0.21	35.09 ± 1.41	1.09 ± 0.40	62%	A2 (C−: *Pleurotus* B.)
0.6 ± 0.30	0.70 ± 0.47	24.40 ± 3.24	3.82 ± 1.31	90%	A3 (C−: δ FeOOH)
0.7± 0.33	0.30 ± 0.15	21.92 ± 3.64	1.86 ± 1.38	66%	A4 (C−: All mat. ^2^)
0.7 ± 0.33	0.60 ± 0.22	24.42 ± 3.70	0.98 ± 0.47	62%	A5 (E2: Banana Peel B.)
0.6 ± 0.30	0.70 ± 0.30	25.70 ± 3.02	1.92 ± 0.74	65%	A6 (E2: *Pleurotus* B.)
1.3 ± 0.39	0.30 ± 0.21	24.38 ± 3.67	1.74 ± 0.74	66%	A7 (E2: δ FeOOH)
0.7 ± 0.21	1.00 ± 0.26	23.26 ± 1.25	1.29 ± 1.06	83%	A8 (E2: All mat. ^2^)
1.3 ± 0.33	0.80 ± 0.55	21.70 ± 3.88	0.95 ± 0.32	57%	A9 (EE2: Banana Peel B.)
0.8 ± 0.25	0.50 ± 0.34	28.24 ± 4.07	2.20 ± 0.68	79%	A10 (EE2: *Pleurotus* B.)
0.9 ± 0.28	0.70 ± 0.26	11.64 ± 0.94	1.22 ± 0.63	79%	A11 (EE2: δ FeOOH)
0.3 ± 0.15	0.60 ± 0.27	18.00 ± 2.29	0.70 ± 0.25	52%	A12 (EE2: All mat. ^2^)

^1^ Standard Error of the Mean. ^2^ All adsorbent materials used in this study. ^3^ Chromosomal Aberrations. ^4^ Micronuclei.

**Table 5:** Mean values of genetic damage from Negative Control, Positive Control, and Hormones before and after the filtration process.

Samples	Level of Genetic Damage					Damage Index and Damage Frequency	
	0	1	2	3	4	DI	DF (%)
C−	68.67	26.67	0	0	0.00	26.67 ± 9.06	26.67
C+	24.00	50.00	20.67	4.00	1.33	108.67 ± 18.70	68.33
E2	91.67	8.33	0.00	0.00	0.00	8.33 ± 1.76	8.33
EE2	79.00	19.00	2.00	0.00	0.00	23.00 ± 9.54	21.00
A1	92.33	7	0.67	0	0	8.33 ± 7.33	7.67
A2	86	14	0	0	0	14.00 ± 6.50	14.00
A3	85	15	0	0	0	15.00 ± 5.86	15.00
A4	80	20	0	0	0	20.00 ± 8.00	20.00
A5	89	11	0	0	0	11.00 ± 10.02	11.00
A6	91	8.66	0.33	0	0	9.67 ± 4.41	9.00
A7	89	11	0	0	0	9.33 ± 4.33	11.00
A8	87	11.67	1	0	0	15.00 ± 3.61	13.00
A9	81.67	17.67	0.67	0	0	19.00 ± 11.93	18.33
A10	78.67	21	0.33	0	0	22.00 ± 4.50	21.33
A11	83.67	14	2.33	0	0	19.00 ± 3.61	16.33
A12	89	11	0	0	0	11.00 ± 5.57	11.00

**Table 6:** Efficiency of removal of E2 and EE2 hormones with different adsorbents reported in other studies.

Reference	Removal (Max.)	Hormone	Adsorbent
Alhares et al. [40]	100.00%	EE2	Rice husk coated with copper oxide nanoparticles
Schmitt; Kieling and Caetano [41]	94.00%	E2	Rice Husk Ash
Schmitt; Kieling and Caetano [41]	96.00%	E2	Activated carbon
Fernandes et al. [42]	76.20%	E2	Decomposed turfa
Fernandes et al. [42]	55.00%	EE2	Decomposed turfa
Prokić et al. [22]	99.20%	E2	Unmodified carbon nanotubes
Prokić et al. [22]	99.54%	EE2	Unmodified carbon nanotubes
Xu et al. [43]	97.08%	EE2	Magnetic MXene composite Fe_3_O_4_@Ti_3_C_2_
Santos et al. [44]	91.70%	EE2	Activated carbon
Zarghi et al. [45]	95.50%	E2	Silica from rice husks
Ferreira et al. [46]	98.00%	E2	Maghemite oxide-graphene nanoparticles
Ferreira et al. [46]	96.00%	EE2	Maghemite oxide-graphene nanoparticles
Zhang and Zhou [47]	99.00%	E2	Granular activated carbon/carbonaceous adsorbent
This study	100%	E2	Banana Peel Biomass, *Pleurotus* Biomass and FeOOH δ
This study	100%	EE2	Banana Peel Biomass, *Pleurotus* Biomass and FeOOH δ

## Data Availability

The raw data supporting the conclusions of this article will be made available by the authors on request.
